# Supplementary material for: TLR7 promotes skin inflammation via activating NFκB-mTORC1 axis in rosacea
Source: PeerJ. 2023 Sep 26;11:e15976. doi: 10.7717/peerj.15976 (PMC10540772; doi:10.7717/peerj.15976)
Supplement: Supplemental Information 1 [file peerj-11-15976-s001.docx]

Supplementary Material

## Supplementary Figures


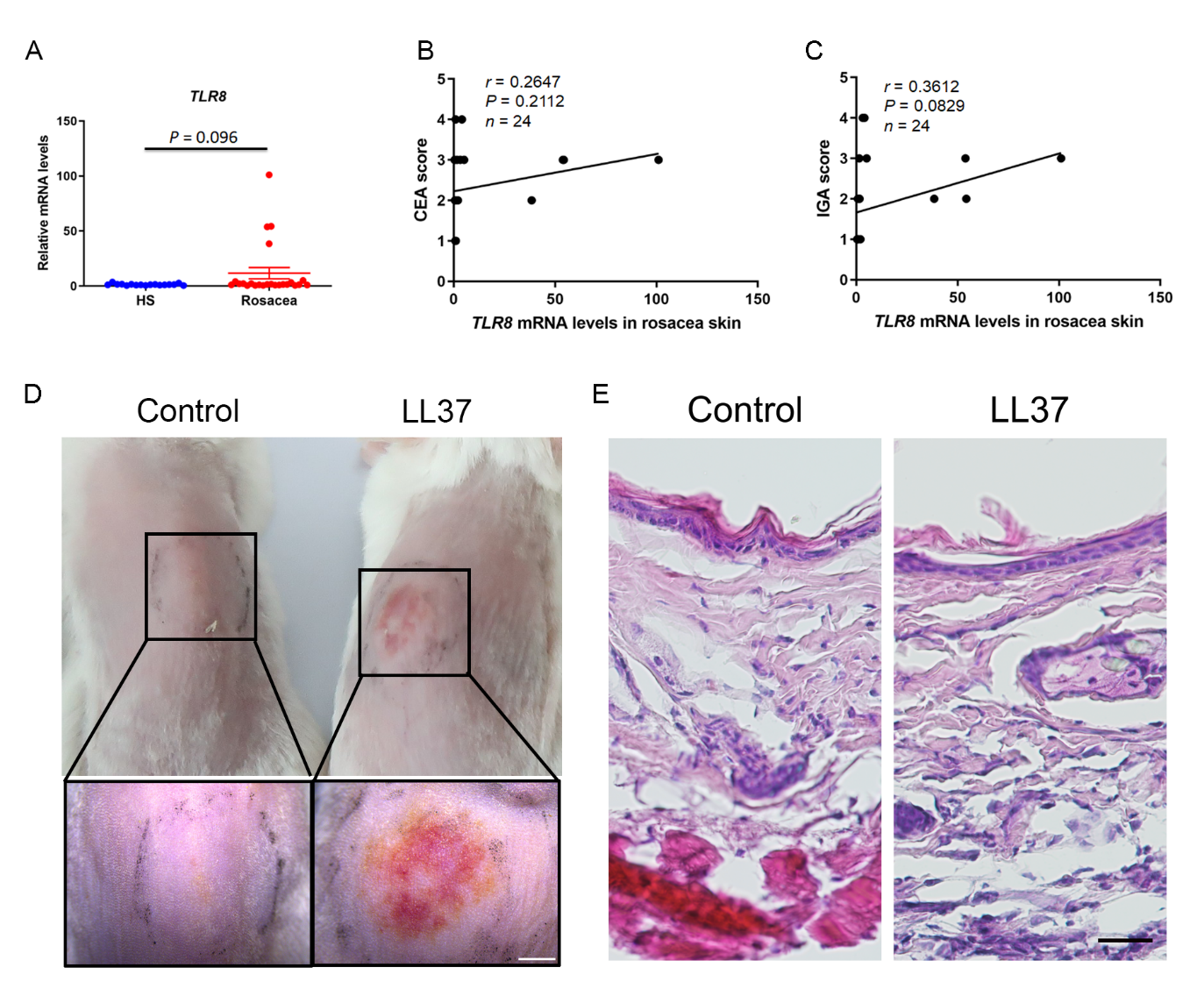


**Supplementary Figure 1.** **(A)** The mRNA expression levels of *TLR8* in the epidermis of human rosacea lesions. HS, *n* = 17; Rosacea, *n* = 24. **(B)** Correlation of *TLR8* mRNA expression in the epidermis of human rosacea lesions (*n* = 24) with the CEA scores. Spearman's correlation coefficient was used for the correlation analysis (two‐tailed). **(C)** Correlation of *TLR8* mRNA expression in the epidermis of human rosacea lesions (*n* = 24) with the IGA scores. Spearman's correlation coefficient was used for the correlation analysis (two‐tailed). **(D)** The back skins of mice were intradermally injected with LL37 or control vehicle (*n* = 6 for each group). Images were taken 48 h after the first LL37 administration. Below panels, magnified pictures of black boxed areas. Scale bar: 2 mm. **(E)** HE staining of lesional skin sections from mice injected with LL37 or control vehicle (*n*= 6 for each group). Scale bar: 200 μm. Data represent the mean ± SEM. ***P* < 0.01.


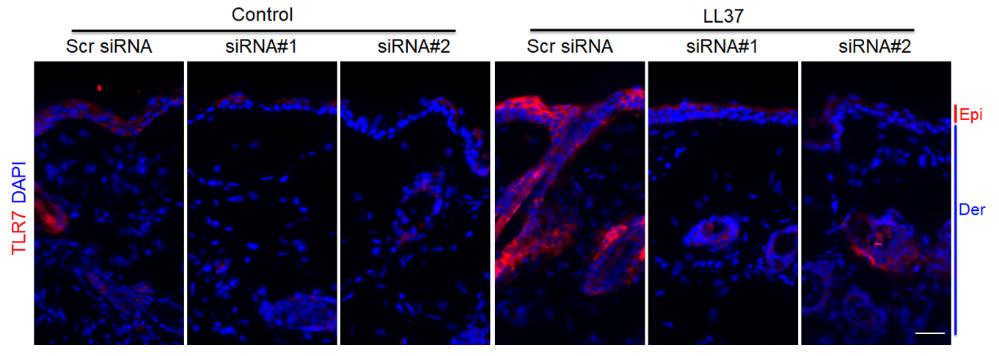


**Supplementary Figure 2.** Immunostaining of TLR7 in skin sections from Scr siRNA and *Tlr7* siRNA mice. Epi, epidermis. Der, dermis. Scale bar: 200 μm. Scr siRNA, scrambled siRNA. All results are representative of at least three independent experiments. Data represent the mean ± SEM. ***P* < 0.01.


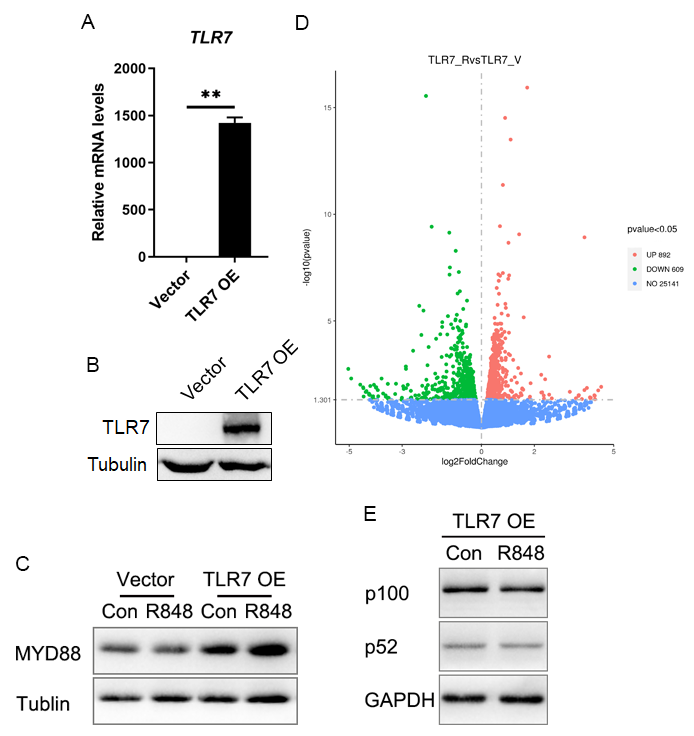


**Supplementary Figure 3.** **(A)** The mRNA expression levels of *TLR7* in primary human keratinocytes transfected with vector or TLR7 (from three replicates). **(B)** Immunoblot analysis of TLR7 in cell lysates from primary human keratinocytes transfected with vector or TLR7. Tublin is the loading control. Data (B) are representative of at least three independent experiments. TLR7 OE, TLR7-overexpression. **(C)** Immunoblot analysis of MYD88 in cell lysates from TLR7-overexpressing primary human keratinocytes treated with R848 for 24 h. Tublin is the loading control. Data (C) are representative of at least three independent experiments. **(D)** In TLR7-overexpressing HaCaT keratinocytes, volcano plot of differential genes between R848 and control after RNA‐sequencing. **(E)** Immunoblot analysis of p100/p52 in cell lysates from TLR7-overexpressing primary human keratinocytes treated with R848 for 15 min. GAPDH is the loading control. Data (E) are representative of at least three independent experiments. All results are representative of at least three independent experiments. Data represent the mean ± SEM. ***P* < 0.01.


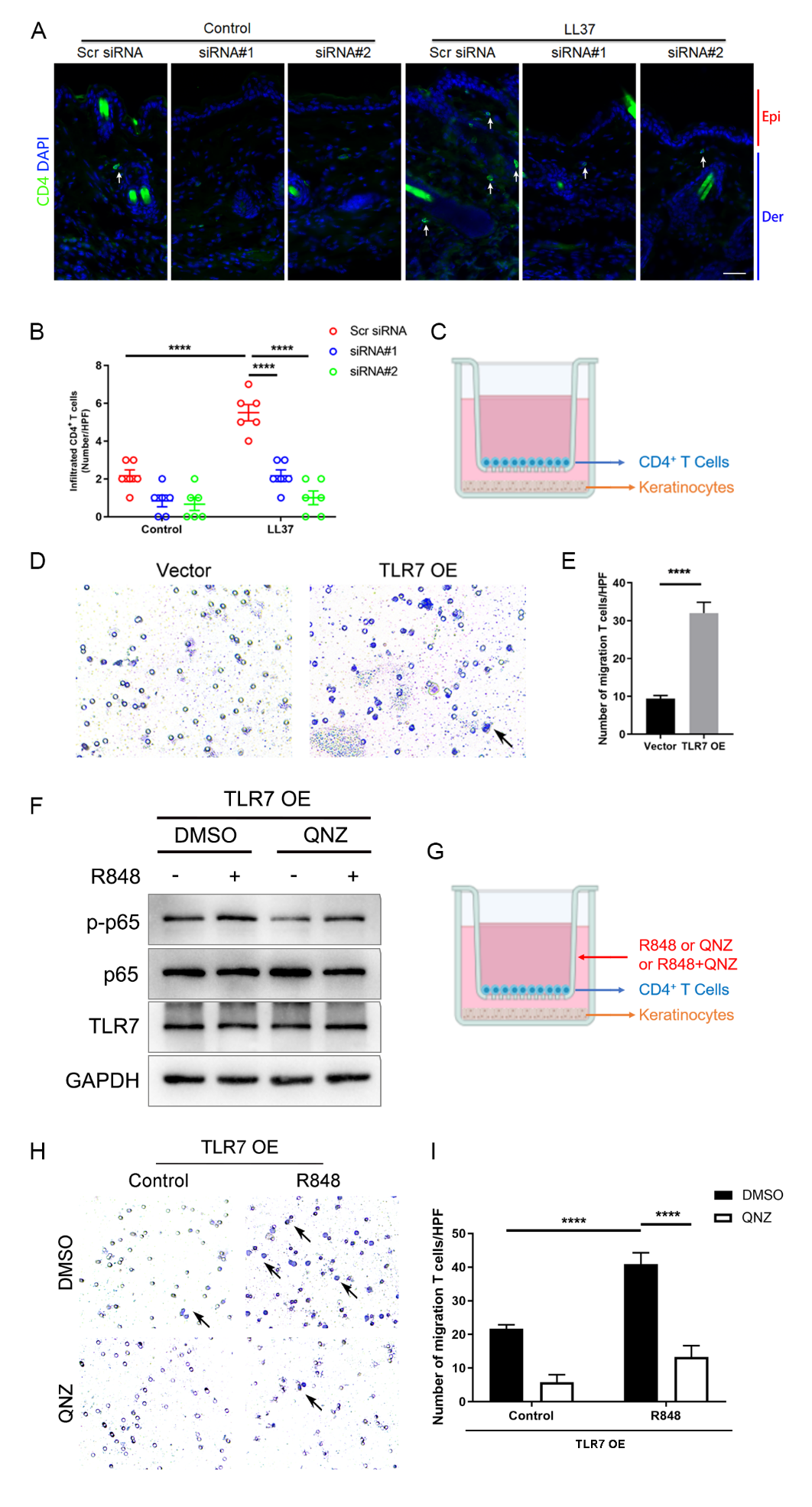


**Supplementary Figure 4.** **(A)** Immunostaining of CD4 in skin sections from Scr siRNA and *Tlr7* siRNA mice. Epi, epidermis. Der, dermis. Scale bar: 200 μm. Scr siRNA, scrambled siRNA. **(B)** Quantification of infiltrated CD4^+^ T cells in dermis (*n* = 6). **(C)** Schematic of the Transwell experiment. Keratinocytes were plated in a well below. Purified CD4^+^ T cells were plated in an 8 μm pore Transwell above. **(D)** Comparison of migration ability of CD4^+^ T cells between the indicated groups (vector or TLR7 OE) based on Transwell assay. TLR7 OE, TLR7-overexpression. **(E)** Quantification of migration T cells. **(F)** Immunoblot analysis of p-p65, total p65 and TLR7 in cell lysates from TLR7-overexpressed primary human keratinocytes treated with QNZ (12 h) and then R848 (15 min). p-p65 protein levels were analyzed relative to total p65. GAPDH is the loading control. Data (F) are representative of at least three independent experiments. **(G)** Schematic of the Transwell experiment. TLR7-expressiong keratinocytes treated with R848 or QNZ were plated in a well below. Purified CD4^+^ T cells were plated in an 8 μm pore Transwell above. **(H)** Comparison of migration ability of CD4^+^ T cells between the indicated groups based on Transwell assay. **(I)** Quantification of migration T cells. All results are representative of at least three independent experiments. Data represent the mean ± SEM. One‐way ANOVA with Bonferroni's *post hoc* test (I) or two‐tailed unpaired Student's *t*‐test (E) was used.


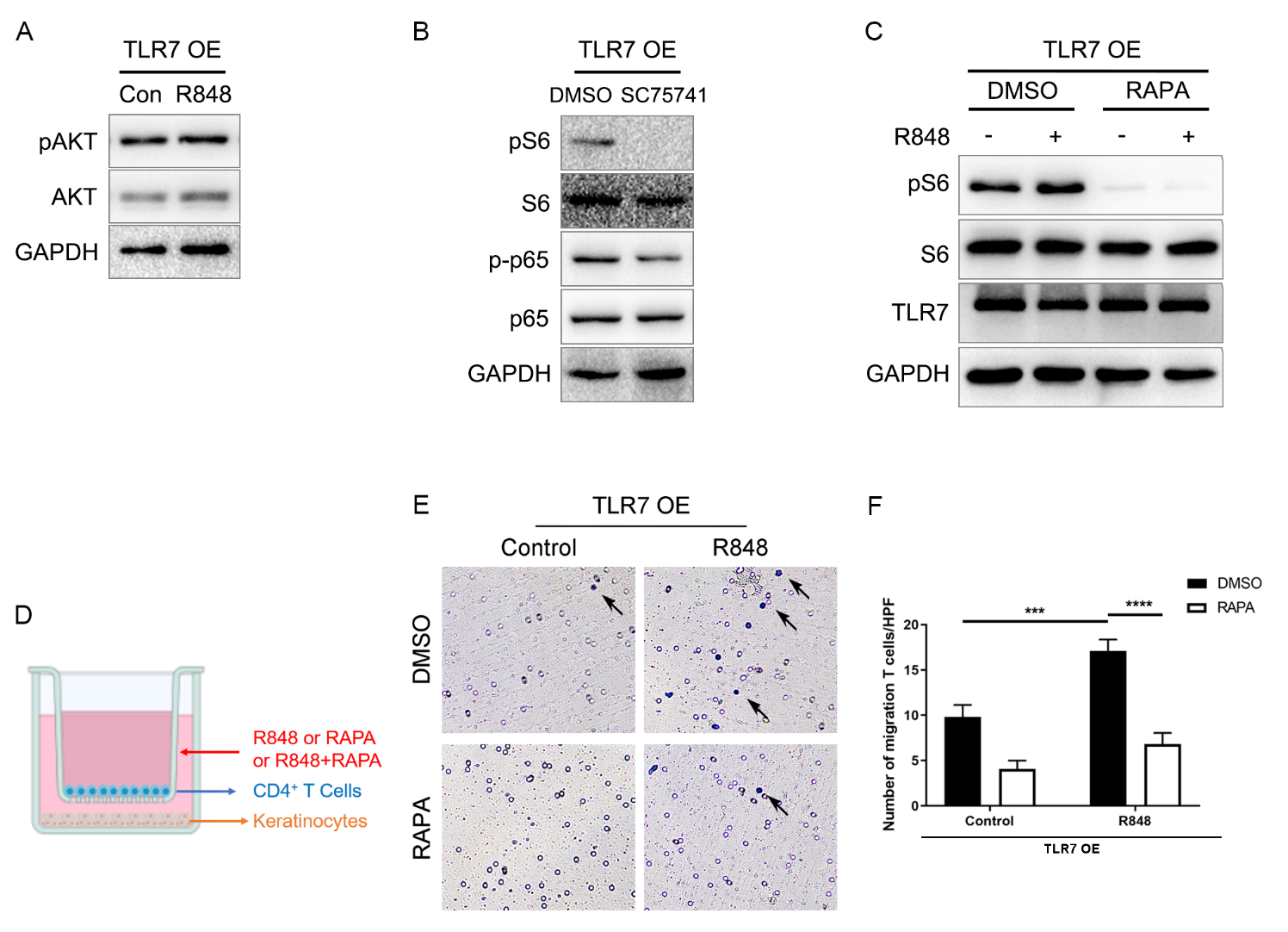


**Supplementary Figure 5.** **(A)** Immunoblot analysis of pAKT and total AKT in cell lysates from TLR7-overexpressing primary human keratinocytes treated with R848 for 15 min. pAKT protein levels were analyzed relative to total AKT. GAPDH is the loading control. Data (A) are representative of at least three independent experiments. TLR7 OE, TLR7-overexpression. **(B)** Immunoblot analysis of pS6, total S6, p-p65, p65 and TLR7 in cell lysates from TLR7-overexpressing primary human keratinocytes treated with SC75741 for 24 h. pS6 and p-p65 protein levels were analyzed relative to total S6 and p65, respectively. GAPDH is the loading control. Data (A) are representative of at least three independent experiments. **(C)** Immunoblot analysis of pS6, total S6 and TLR7 in cell lysates from TLR7-overexpressed primary human keratinocytes treated with RAPA (2 h) and then R848 (15 min). Ps6 protein levels were analyzed relative to total S6. GAPDH is the loading control. Data (C) are representative of at least three independent experiments. **(D)** Schematic of the Transwell experiment. TLR7-expressiong keratinocytes treated with R848 or RAPA were plated in a well below. Purified CD4^+^ T cells were plated in an 8 μm pore Transwell above. **(E)** Comparison of migration ability of CD4^+^ T cells between the indicated groups based on Transwell assay. **(F)** Quantification of migration T cells. All results are representative of at least three independent experiments. Data represent the mean ± SEM. One‐way ANOVA with Bonferroni's *post hoc* test (F) was used.
