# Supplementary material for: TLR7 promotes skin inflammation via activating NFκB-mTORC1 axis in rosacea
Source: PeerJ. 2023 Sep 26;11:e15976. doi: 10.7717/peerj.15976 (PMC10540772; doi:10.7717/peerj.15976)

**Full unedited blot for Figure 3C**

**
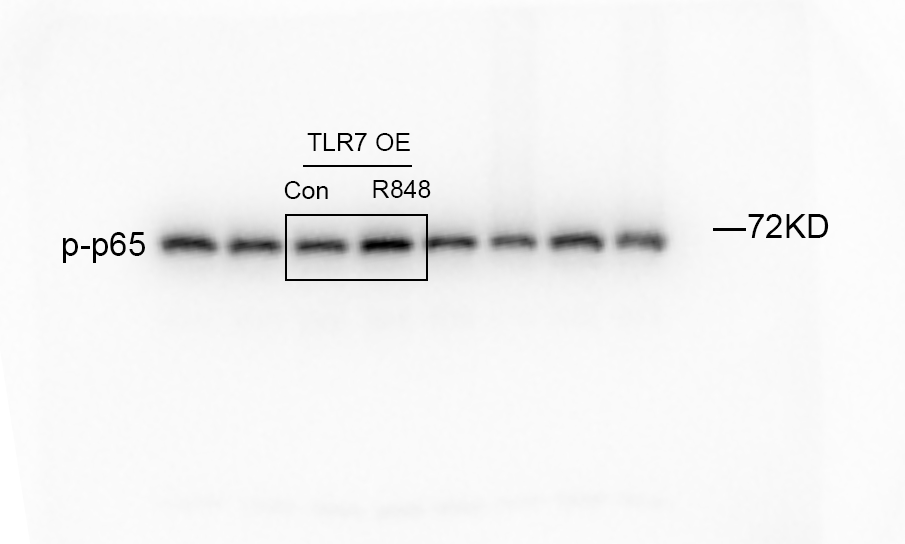

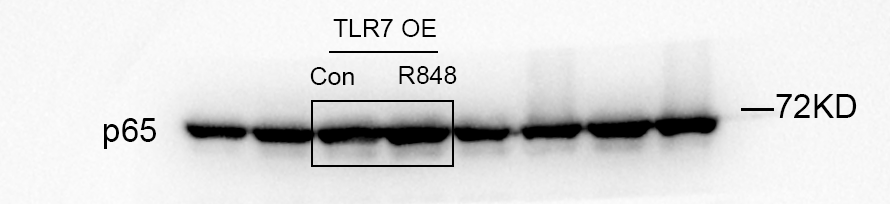

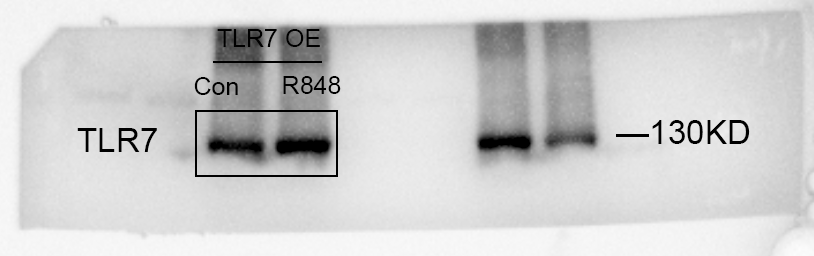

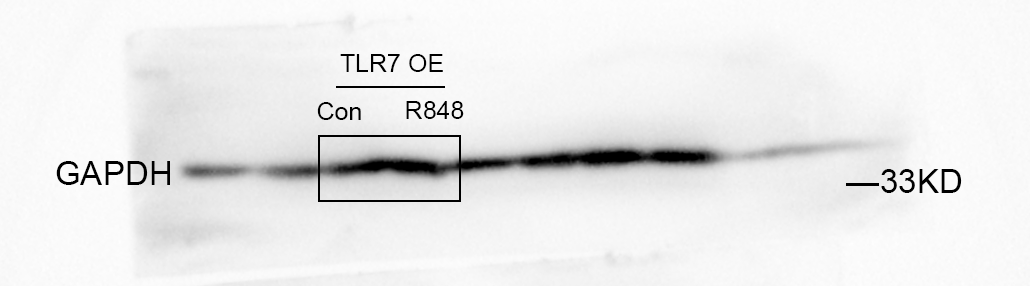
**

**Full unedited blot for Figure 5C**

**
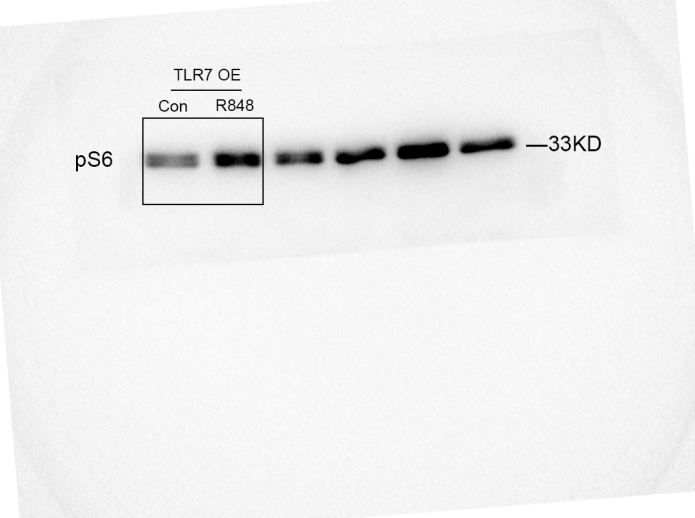

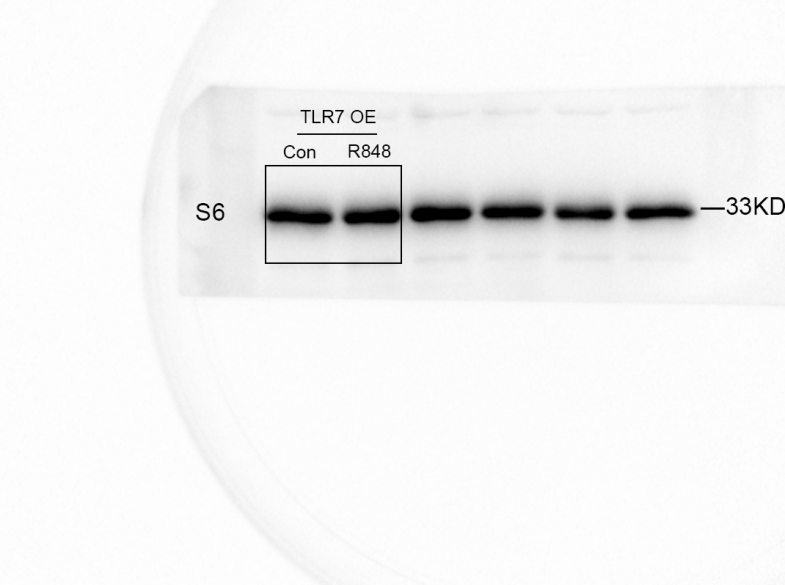

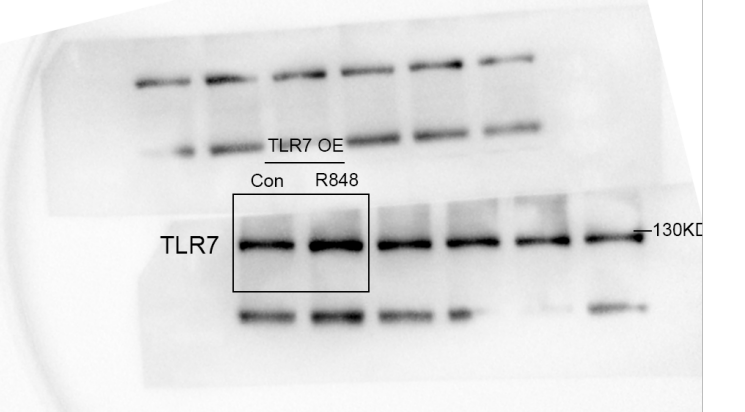

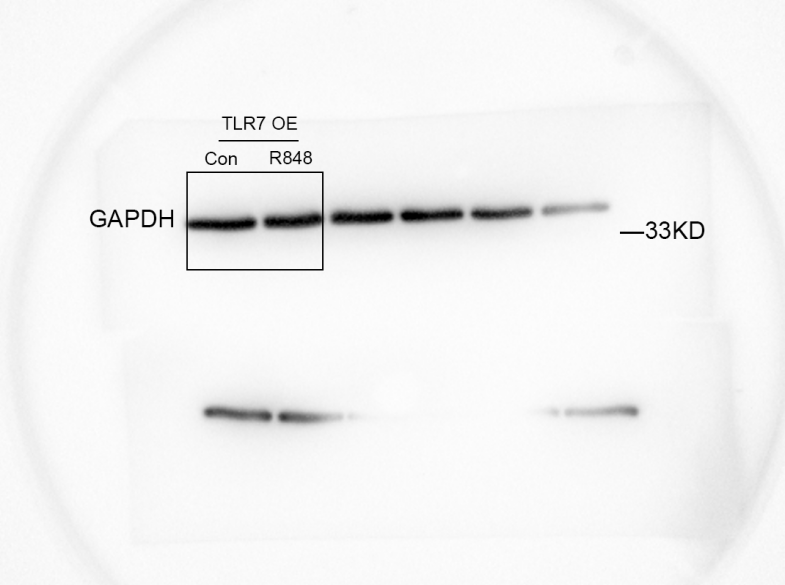
**

**Full unedited blot for Figure 5D**

**
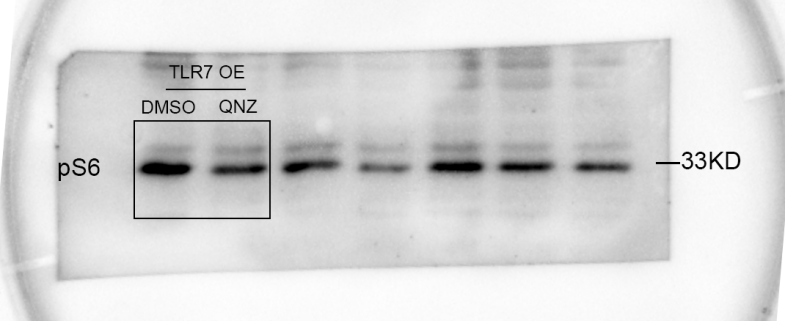

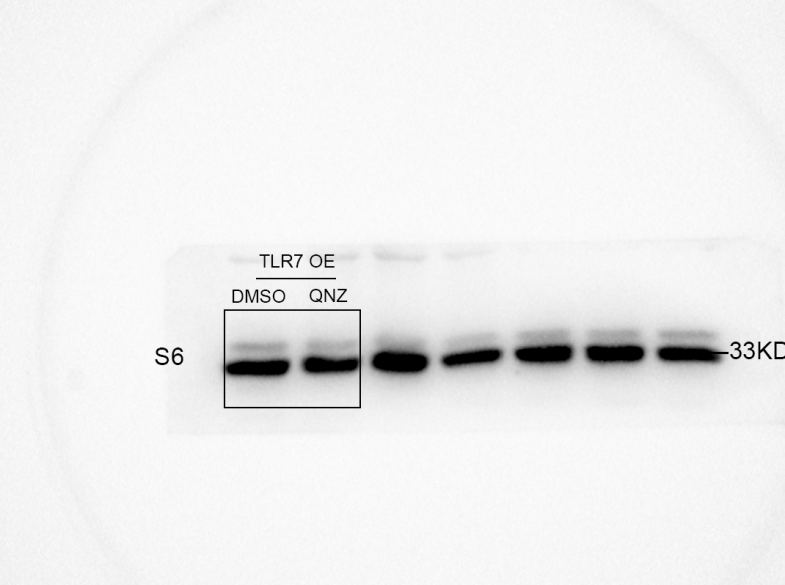

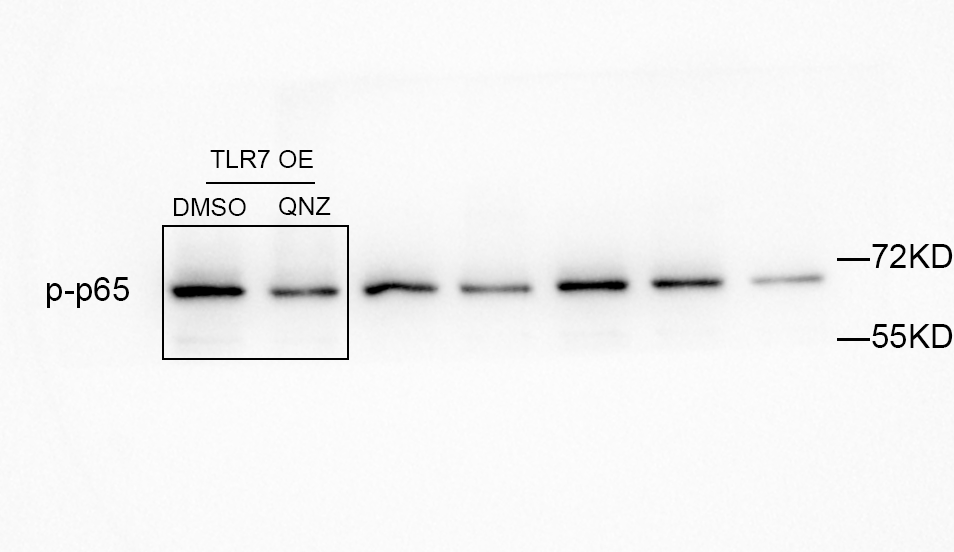

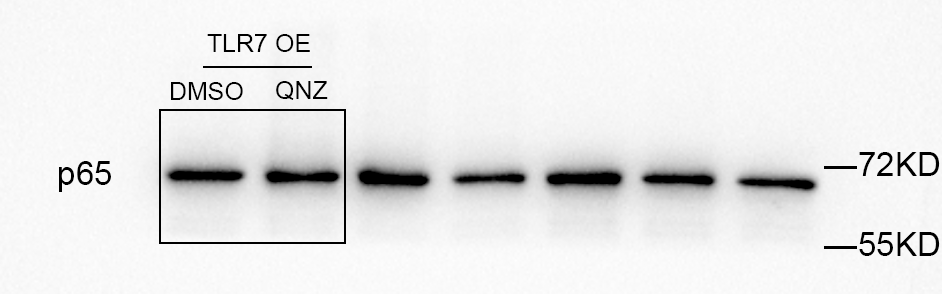

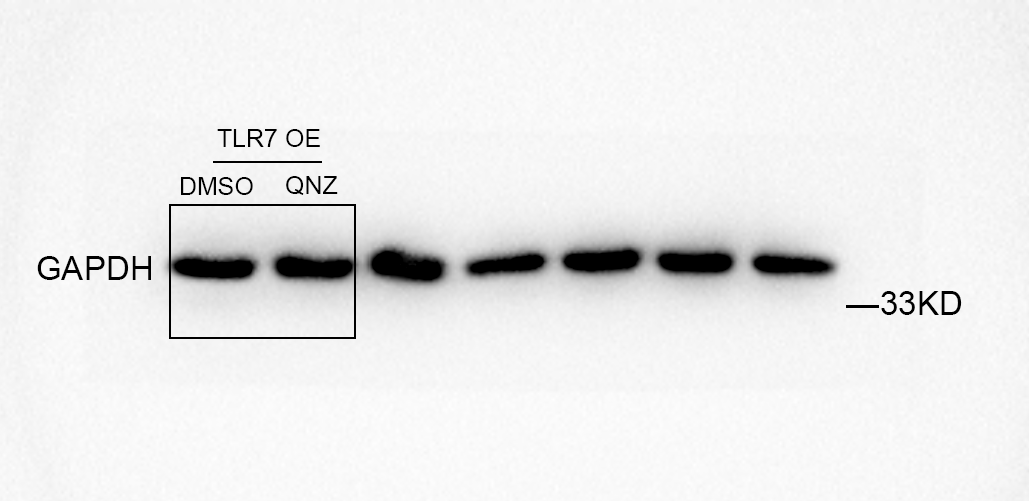
**

**Full unedited blot for Figure 5E**


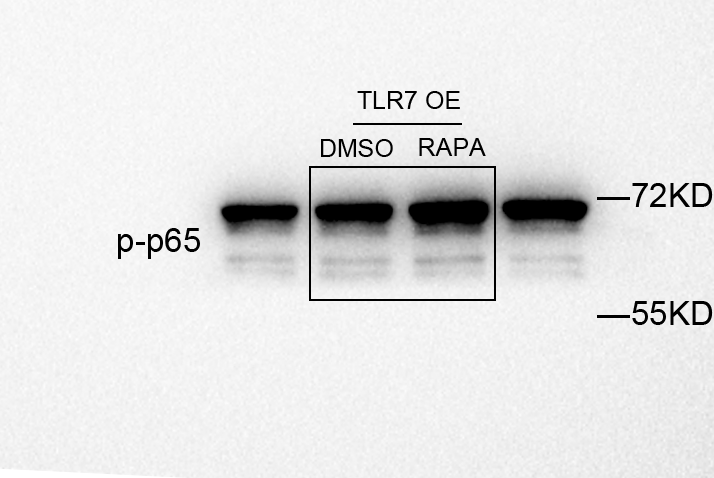

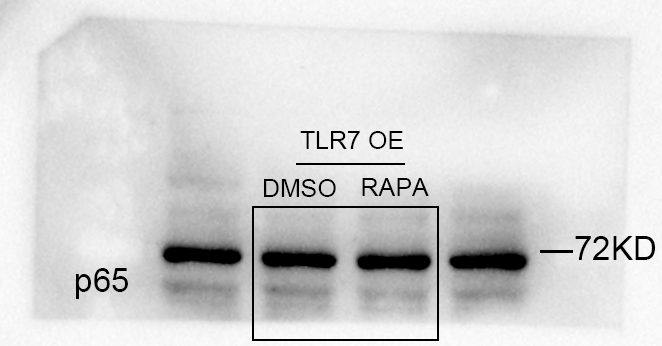

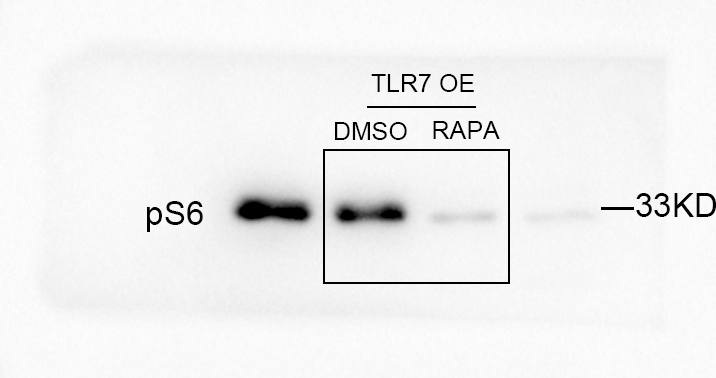

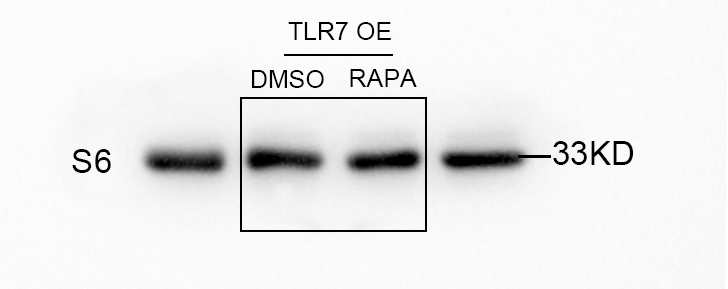

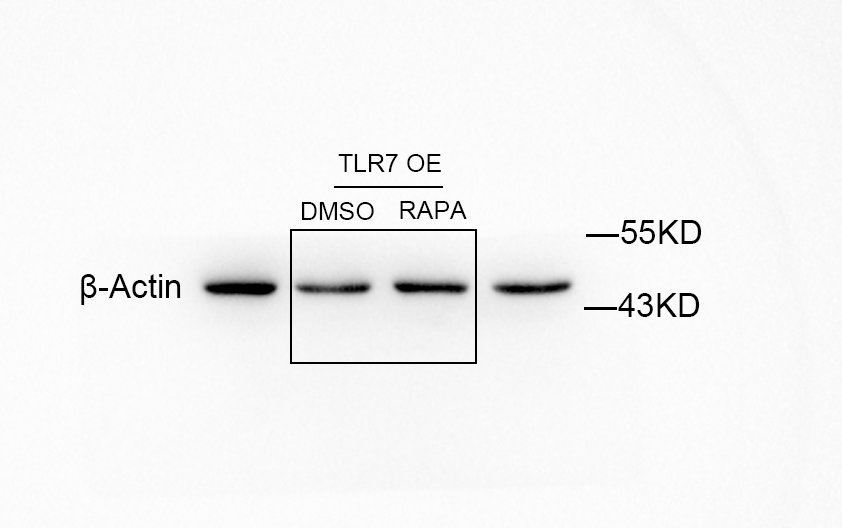

Supplement: Supplemental Information 2 [file peerj-11-15976-s002.docx]
